# Time Course of Changes in Serum Oxidative Stress Markers to Predict Outcomes for Surgical Treatment of Lumbar Degenerative Disorders

**DOI:** 10.1155/2020/5649767

**Published:** 2020-12-24

**Authors:** Hiroshi Takahashi, Yasuchika Aoki, Junya Saito, Arata Nakajima, Masato Sonobe, Yorikazu Akatsu, Shinji Taniguchi, Manabu Yamada, Keita Koyama, Yuki Akiyama, Yasuhiro Shiga, Kazuhide Inage, Sumihisa Orita, Yawara Eguchi, Satoshi Maki, Takeo Furuya, Tsutomu Akazawa, Masao Koda, Masashi Yamazaki, Seiji Ohtori, Koichi Nakagawa

**Affiliations:** ^1^Department of Orthopaedic Surgery, Toho University Sakura Medical Center, 564-1 Shimoshizu, Sakura City, Chiba 285-8741, Japan; ^2^Department of Orthopaedic Surgery, Faculty of Medicine, University of Tsukuba, Ibaraki, Japan; ^3^Department of Orthopaedic Surgery, Eastern Chiba Medical Center, Chiba, Japan; ^4^Department of Orthopaedic Surgery, Chiba University Graduate School of Medicine, Chiba, Japan; ^5^Department of Orthopaedic Surgery, St. Marianna University School of Medicine, Kanagawa, Japan

## Abstract

Recent reports indicate that oxidative stress is involved in the pathobiology of acute spinal cord injury or compression myelopathy. We conducted an observational study to determine levels of oxidative stress markers in serum from 80 patients who underwent spinal surgery to treat neurological symptoms related to lumbar degenerative disorders. Serum samples were collected before surgery and at 3 months, 6 months, and 1 year after surgery. Derivatives of reactive oxygen metabolites (ROM) in the serum samples were measured to gauge the level of oxidative stress. For preoperative neurological evaluation, patients were assessed for motor weakness in the lower extremities. We divided the patient samples into two groups: ROM decreasing at 1 year after surgery (G group) and ROM increasing at 1 year after surgery (W group). Then, we evaluated clinical outcomes using the visual analog scale and Oswestry disability index (ODI). Among the samples from the 80 enrolled patients, mean ROM levels before surgery increased to 388.5 ± 92.0, indicating the presence of moderate oxidative stress. The level of ROM gradually decreased after surgery and 1 year after surgery: the levels had significantly decreased to 367.6 ± 83.3 (*p* < 0.05). In patients who exhibited motor weakness, ROM values were significantly increased compared to those patients who had no motor weakness (*p* < 0.05). In analyses of clinical outcomes, ODI values for the W group 1 year after surgery were significantly higher than those for the G group (*p* < 0.05). Moderate oxidative stress was present in patients who had lumbar degenerative disorders and the degree of oxidative stress gradually improved within 1 year after surgery. The clinical results suggest that neurogenic oxidative stress can be mitigated by surgery for patients with lumbar degenerative disorders, and residual oxidative stress reflects poor surgical outcomes.

## 1. Introduction

Among degenerative lumbar disorders, lumbar spinal stenosis (LSS) and lumbar disc herniation (LDH) are the most common. The mechanism of LSS is related to chronic compression of the cauda equina and/or nerve root caused by thickening of the ligamentum flavum, degenerated discs, or osteophytes. Spinal surgery is needed for patients with LSS and LDH who do not respond to conservative treatment. Rapid progression of motor paralysis such as foot drop occasionally accompanies LDH or even LSS that usually results in a slow and stepwise decline in function. In such cases, early surgical treatment is recommended as the only valid therapy. However, some patients have poor surgical outcome and do not achieve sufficient improvement in neurological function [1]. In addition, residual low back pain or symptoms involving lower extremities that are associated with failed back surgery syndrome despite successful surgical decompression or fusion can become problematic [2]. Currently, there is no method that allows the accurate prediction of the recovery of neurological function or the degree of pain relief before surgical treatment. Therefore, several biomarkers to predict the degree of pain relief or damage to the cauda equina and nerve root would be valuable.

Recent reports indicate that oxidative stress markers in serum are elevated in various neurodegenerative disorders such as Alzheimer's disease, amyotrophic lateral sclerosis, and Parkinson's disease [3–5]. For spinal lesions, levels of serum oxidative stress markers are elevated in patients with acute spinal cord injury or compression myelopathy [6–8]. In particular, a recent report indicated that increased amounts of serum oxidative stress influence neurological recovery after surgery and manifest as acute worsening of symptoms of cervical compression myelopathy [8]. However, to our knowledge, no studies have analyzed the time course of changes in serum oxidative stress markers before and after surgery in patients with lumbar degenerative disorders. Here, we conducted an observational study to determine the levels of oxidative stress markers in serum samples from patients with lumbar degenerative disorders (LSS and LDH) and to determine whether there is a relationship between levels of oxidative stress markers, patient condition prior to surgery, and clinical outcome.

## 2. Materials and Methods

### 2.1. Patient Selection

This study was approved by the Human Ethics Committee at Toho University Sakura Medical Center. Informed consent was obtained from all patients. A total of 150 patients who underwent spinal surgery to treat neurological symptoms due to lumbar degenerative disorders in our hospital between April 2015 and September 2018 were recruited for this study. All patients underwent adequate conservative treatment or had an operative indication such as foot drop or bladder disturbance. Exclusion criteria included patients from whom consent could not be obtained (3 patients); patients who were younger than 20 years old (1); patients who were diagnosed with spinal cord tumor (2); and patients who had infection (4), trauma (including minor trauma, 9), deformity (3), neurodegenerative diseases (2), rheumatoid arthritis (4), malignant disease of organs (5), or a double lesion (e.g., cervical or thoracic compression myelopathy with lumbar degenerative disorders) (5). Another 9 patients who dropped out during follow-up and 23 patients for whom sample volumes were insufficient to perform all assays were also excluded. Ultimately, 80 patients were enrolled, and of these, 14 and 66 had LDH and LSS, respectively, which were diagnosed by three orthopaedic spine surgeons based on neurological findings, X-ray, and magnetic resonance images. All patients were followed up for at least 1 year after surgery.

### 2.2. Measurement of Serum Oxidative Stress Markers

A recently developed method for measuring derivatives of reactive oxygen metabolites (ROM) in serum, referred to as the d-ROM test, was used to evaluate reactive oxygen species (ROS) production that was not counteracted by antioxidative activity and thus was representative of oxidative stress in serum. The d-ROM test was performed using the integrative Free Radical Analytical System (FRAS, Wismarl, Italy) according to the manufacturer's specifications [9, 10]. The d-ROM test does not measure ROS directly, but rather detects hydroperoxide metabolites that are the main derivatives of ROM. Hydroperoxides are converted into radicals that oxidize N,N-diethyl-*para*-phenylenediamine and can be detected spectrophotometrically using an automatic analyzer. ROM are expressed in arbitrary units termed Carratelli units (U.CARR). For patients who exhibited oxidative stress, ROS produced in serum could be measured in terms of ROM levels. The normal range for ROMs is typically 250–300 U.CARR, whereas ROM levels for moderate and severe oxidative stress corresponded to >340–400 U.CARR and >400 U.CARR, respectively [8, 10]. All serum samples were collected during hematological examinations at about one month before surgery and at 3 months, 6 months, and 1 year after surgery. As background parameters, the relationship between ROM and patient age, body mass index (BMI), sex, history of smoking, and existence of diabetes were examined.

### 2.3. ROM and Surgical Stress

All surgeries for the enrolled patients were planned and performed by three orthopaedic spine surgeons using a posterior approach. All decompression surgeries were performed using unilateral laminectomy for bilateral decompression. All fusion surgeries were performed using transforaminal lumbar interbody fusion via a hemiopen approach with a percutaneous pedicle screw system using guide wire to minimize damage to paraspinal muscles. For lumbar discectomy, surgeries were performed using a tubular retractor (METRx MD system, Medtronic, US) to further minimize invasive damage to paraspinal muscles. The corresponding author was involved in all the surgeries as the surgeon or the leading assistant, and all the surgical procedures (decompression and fusion) were standardized in the damage to paraspinal muscles. The relationship between ROM and surgical level (i.e., single level or multiple level) was examined, as was the relationship between ROM and surgical procedure type (i.e., decompression surgery or fusion surgery).

### 2.4. ROM and Neurological Factors

The neurological status of all patients was evaluated by three orthopaedic spine surgeons. First, the relationship between ROM and the cause of disorder (LSS or LDH) was investigated. Second, neurological evaluation of the existence or absence of motor weakness in the lower extremities was assessed.

### 2.5. ROM and Clinical Outcome

Visual analog scale (VAS; 100 mm) scores for low back pain (LBP), lower extremity pain (LEP), and lower extremity numbness (LEN) as well as the Oswestry disability index (ODI) were measured as clinical outcomes [11]. The definition of LBP in this study excluded buttock pain. All VAS and ODI values were measured before surgery and 1 year after surgery. Samples were divided into two groups: the G group and the W group for which levels of ROM decreased and increased, respectively, 1 year after surgery. Differences between the two groups were evaluated.

### 2.6. Statistical Analyses

Results are expressed as the mean ± standard deviation (SD). A repeated-measure two-factor ANOVA with a post hoc Tukey-Kramer test and paired *t*-test were used to evaluate the time course of changes in serum ROM. A Student's *t*-test and one-factor ANOVA with a post hoc Tukey-Kramer test were used to evaluate the relationship between ROM and each parameter. *p* < 0.05 was considered statistically significant. All statistical analyses were performed using SPSS software (Version 21, IBM Corporation, Armonk, NY, USA).

## 3. Results

### 3.1. Patients and Characteristics

Patient characteristics are shown in Table 1. The preoperative mean ROM value of 388 U.CARR indicated the presence of moderate oxidative stress in the overall patient population. There was no bias in the ratio of males and females. LSS tended to be more frequent than LDH but the difference in frequencies was not significant. Among the 80 enrolled patients, 10 smoked and 19 had diabetes. The numbers of decompression and fusion surgeries did not differ significantly. The average amount of bleeding during surgery is less than one hundred grams, and there were no cases that required blood transfusion. In addition, there were no cases that had pain recurrence (and required revision surgery) due to the recurrence of disc herniation or other segment disorders including adjacent segment degenerations within one-year follow-up.

### 3.2. The Time Course Changing of ROM in Serum

The levels of ROM gradually decreased after surgery, and 1 year after surgery, the average value had significantly decreased to 367.6 ± 83.3 U.CARR compared with that before surgery (*p* < 0.05; Table 2). Based on these results, we evaluated the relationship between ROM and each parameter before and 1 year after surgery.

### 3.3. Relationship between ROM and Background Parameters

**Figure 1:**
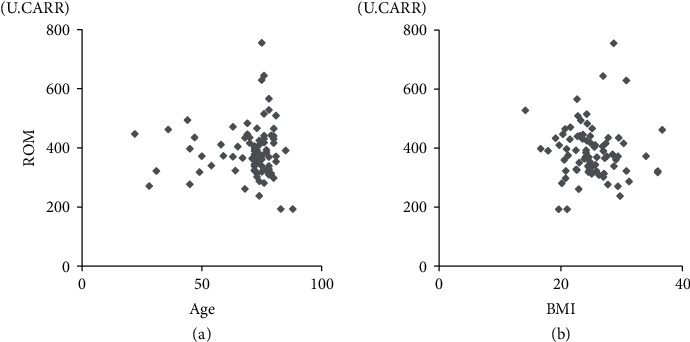
Relationship of reactive oxygen metabolite (ROM) levels with age and body mass index. Relationship between reactive oxygen metabolites (ROM) and (a) age and (b) body mass index (BMI).

**Figure 2:**
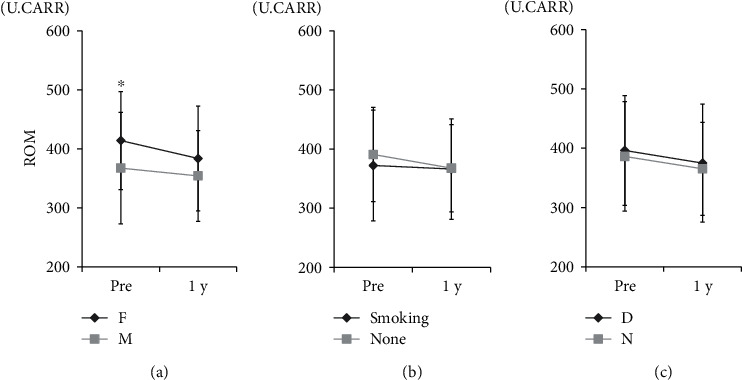
Relationship of reactive oxygen metabolite (ROM) levels with sex, smoking status, and diabetes. Relationship of ROM with (a) sex (F: female; M: male; ^∗^*p* < 0.05 for comparisons using Student's *t*-test); (b) smoking status; (c) presence of diabetes (D: history of diabetes: N: no history of diabetes).

We next investigated the relationship between ROM and background parameters that could influence ROM levels. There was no significant correlation between ROM and age or BMI (Figure 1). Meanwhile, serum ROM levels among female patients were significantly higher than those for males before surgery (*p* < 0.05; Figure 2). There was no significant difference in the timeline for improvement in levels of ROM based on sex, smoking, or presence of diabetes.

### 3.4. Relationship between ROM and Surgical Stress

**Figure 3:**
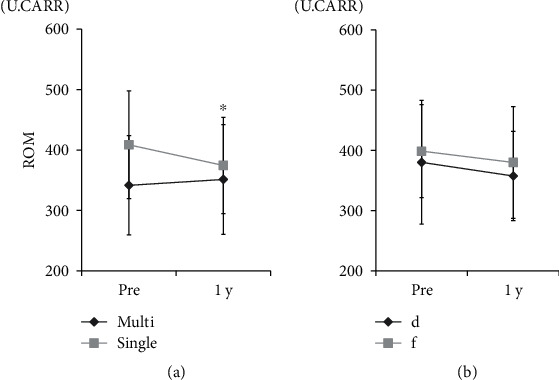
Relationship of reactive oxygen metabolite (ROM) levels with surgical level and procedure type. ROM and (a) surgical type (^∗^*p* < 0.05 for comparisons using a repeated-measure two-factor ANOVA) and (b) surgical procedure (d: decompression; f: fusion).

In comparing changes in ROM between single or multiple surgical levels as well as surgical procedure type (decompression or fusion), a significant improvement in levels of ROM was observed for single-level surgery compared to that for multiple-level surgery (*p* < 0.05), but no significant difference in ROM levels was seen between decompression and fusion surgery (Figure 3).

### 3.5. Relationship between ROM and Neurological Factors

**Figure 4:**
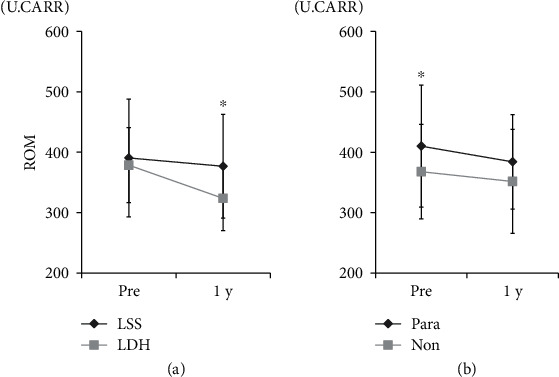
Relationship of reactive oxygen metabolite (ROM) levels with lumbar degenerative disorder diagnosis and with motor weakness. ROM and (a) diagnosis (LSS: lumbar spinal stenosis; LDH: lumbar disc herniation; ^∗^*p* < 0.05 for comparisons using a repeated-measure two-factor ANOVA) and (b) existence of motor weakness (^∗^*p* < 0.05 for comparisons using Student's *t*-test).

In terms of disorder cause, patients with LDH exhibited better improvement in ROM levels compared to those with LSS (*p* < 0.05; Figure 4(a)). A significant increase in ROM levels before surgery was observed for patients displaying motor weakness (*p* < 0.05), but no significant difference in the degree of improvement of ROM was seen between patients who did and did not have motor weakness (Figure 4(b)).

### 3.6. Relationship between ROM and Clinical Outcome

**Figure 5:**
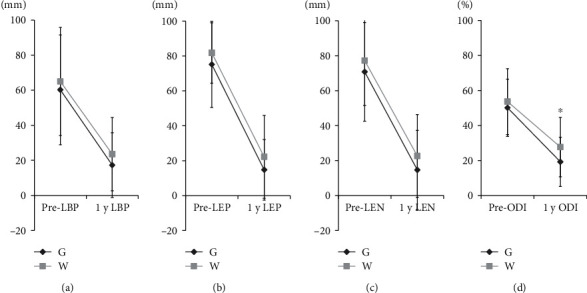
Relationship of reactive oxygen metabolite (ROM) levels with changes in disease indices. ROM and change in (a) LBP (low back pain); (b) LEP (lower extremity pain); (c) LEN (lower extremity numbness); (d) Oswestry disability index (ODI). ^∗^*p* < 0.05 for comparisons using repeated-measure two-factor ANOVA.

Similar and significant improvements in VAS for LBP, LEP, and LEN were observed for both the G and W groups (Figure 5). On the other hand, ODI values for patients in the W group at 1 year after surgery were significantly higher than those for patients in the G group (*p* < 0.05).

## 4. Discussion

In this study, we described the time course for changes in levels of serum ROM as a marker of serum oxidative stress in patients who underwent surgery to treat lumbar degenerative disorders. To the best of our knowledge, this is the first study to examine the time course for postoperative changes of serum ROM in lumbar degenerative disorders, which is a recognized proxy for reactive oxygen species production and has been used to evaluate oxidative stress levels associated with multiple diseases [4, 5, 12]. In this study, we excluded patients who had a history of rheumatoid arthritis or neurodegenerative diseases that could have elevated serum oxidative stress [5, 12].

In the cardiovascular field, several studies investigated the time course of changes in levels of serum oxidative stress markers. Christen et al. reported that serum oxidative stress markers were elevated after surgery, and these levels returned to normal values within a few days after surgery [13]. On the other hand, Kanaoka et al. reported that ROM levels peaked 3 weeks after surgery and the increased levels persisted for 3 months after surgery [14]. Ischemia reperfusion injury is proposed to be one of the main mechanisms for the persistence of elevated levels of oxidative stress markers after cardiovascular surgery [15]. In this study, we found that levels of ROM were moderately elevated before lumbar spinal surgery, and these levels gradually improved during the 1 year after surgery, reflecting the slow time course of improvements. In fact, the degree of ROM improvement we observed was smaller than that seen for the treatment of rheumatoid arthritis [12]. There are several possible explanations for this observation. First, improvements in neurogenic oxidative stress that are associated with neuronal apoptosis that occurs in response to acute spinal cord injury or compression myelopathy may occur slowly. Second, ischemia reperfusion injury after decompression of cauda equina nerves or nerve roots may promote oxidative stress during the early postoperative phase, as was previously reported for surgical outcomes to treat compression myelopathy [16]. Third, upregulation of endogenous antioxidants may occur, as was previously reported [17]. Fourth, the pain relief afforded by surgery may allow patients to gradually resume activities of daily living, and the time needed to recover lost muscle mass may be associated with the more gradual improvement in ROM levels. Indeed, significant improvement in ROM levels was seen for single-level surgery relative to multiple-level surgery in which involvement of multifidus muscles is greater than that for single-level surgery. Recent reports indicated that invasion of paraspinal muscles can influence elevations in oxidative stress markers [18, 19]. On the other hand, the surgical invasion like the surgical time or the amount of bleeding during surgery may also affect the changes of ROM levels after surgery. In lumbar spinal surgery, although surgical time per single-level decompression or fusion was almost constant, multiple-level surgery obviously takes more time. We evaluated the surgical levels (single-level surgery versus multiple-level surgery) that also reflect the surgical time. The result showed that significant improvement in ROM levels was seen for single-level surgery relative to multiple-level surgery. Thus, a longer operative time may also affect the changes of ROM levels. On the other hand, generally, the bleeding during surgery is not massive in this type of lumbar spinal surgery. In this study, we excluded the cases of deformity that required thoracolumbar long fusion that sometimes cause massive bleeding and require blood transfusion to eliminate the effect of operative invasion to ROM levels. In fact, the average amount of bleeding was less than one hundred grams in this study, and there were no cases that required blood transfusion. Thus, the bleeding during surgery have less effect to the changes of ROM levels after surgery in this study. Additional basic and clinical studies will be needed to define in greater detail the mechanisms involved in the observed improvement in ROM levels.

Markers of oxidative stress were previously shown to increase with increasing age, and higher levels were also associated with female gender, obesity, smoking, and diabetes as well as with endogenous oxidative stress [10, 20]. Here, we showed that levels of ROM were increased in females, a finding that is in part consistent with that of a previous study [8]. However, there was no significant correlation between ROM levels and age, BMI, smoking status, and diabetes. These endogenous factors may not have a substantial relationship with ROM at least in lumbar degenerative disorders like LSS or LDH. Meanwhile, the level of oxidative stress based on serum analyses was shown to increase in patients with acute spinal cord injury, compression myelopathy, Alzheimer's disease, amyotrophic lateral sclerosis, or Parkinson's disease [4–8]. Reports of oxidative stress in the presence of neurodegenerative diseases suggest the involvement of neuronal cell apoptosis. In this study, patients with LDH had better improvement in levels of ROM compared to those with LSS. Patients with LDH typically show relatively acute compression of the cauda equina nerves or nerve roots compared to those with LSS, which could explain the increased improvement in ROM levels seen for patients with LDH. In addition, a significant increase in levels of ROM before surgery was observed for patients who exhibited motor weakness, which could reflect how the severity of neuronal damage influences serum oxidative stress. In the assessment of clinical outcome, ODI values for the W group at 1 year after surgery were significantly higher than those for the G group, indicating that residual ROM could indeed be associated with poor surgical outcome. Together, the results of the present study suggest that neurogenic oxidative stress could be improved by decompression (or fusion) surgery in lumbar degenerative disorders like LSS or LDH and that residual oxidative stress reflects surgical outcomes. However, the ratio of endogenous oxidative stress to neurogenic oxidative stress is unclear, as is whether endogenous or neurogenic factors are strongly influential in lumbar degenerative disorders. Further basic and clinical investigations to clarify the mechanism by which oxidative stress increases in lumbar degenerative disorders are needed to guide selection of antioxidant medications or nutritional antioxidant treatments that could improve surgical outcomes.

The present study has some limitations. First, the lack of parameters to evaluate the antioxidant condition like a biological antioxidant potential test is a major limitation of the present study. It was reported that the balance of oxidant and antioxidant conditions was important in other neurodegenerative disorders like Alzheimer's disease [4]. In the present study, as the explanation for the gradual improvement of ROM after surgery, the upregulation of endogenous antioxidants may occur as previously mentioned [17]. In addition, the degree of neurogenic antioxidant condition is unclear. Further investigations of the antioxidant condition will be expected to clarify the detailed mechanism of oxidative stress changes in lumbar degenerative disorders. Second, we had no data for patients who underwent conservative treatments. In cases of surgical treatment, hematological examinations are needed to define the general status and presence of surgical site infection. However, ethical considerations preclude such examinations of patients who underwent conservative treatment. Third, we did not evaluate imaging findings from procedures such as magnetic resonance imaging (MRI). In this study, we focused on changes in neurogenic environment (e.g., decompression of cauda equina nerves or nerve roots) with changes in levels of ROM. In the lumbar nerve root evaluation, a recent report indicated the advance of diffusion tensor imaging (DTI) and diffusion-weighted magnetic resonance (MR) neurography based on MRI as the imaging biomarker [21]. However, for the detailed imaging evaluation using DTI or MR neurography, 3 T MRI is required. In this study, all the patients underwent MRI before and after surgery to confirm that cauda equina or nerve root decompression was sufficient. However, we could not use 3 T MRI for all the patients because of a hardware problem. In addition, it is impossible to have a detailed evaluation of MRI like DTI after surgery in patients who underwent the posterior instrumented and fusion surgery because of implant halation. For these reasons, we excluded the MRI evaluation. Future investigation to evaluate the relationship between the MRI findings and ROM will be needed. In addition, a recent report indicated that modic type I endplate changes in lumbar spine vertebrae can influence elevations in oxidative stress [22]. In the present study, no cases were complicated by modic type I change. As such, further investigation will be needed to characterize how endplate condition affects ROM levels. Fourth, serum oxidative stress markers can be influenced not only by endogenous or neurogenic factors but also by osteoporosis, loss of muscle volume including sarcopenia, or other segment lumbar degenerative disorders. In this study, the data of bone mineral density before surgery is lacking because of the ethical problem that we could not investigate the bone mineral density in young patients. This is one of the major limitations of this study. We excluded the cases complicated with osteoporotic vertebral fracture to minimize the effect of predominantly osteoporosis to ROM. On the other hand, advanced age may cause osteoporosis and affect the change of ROM. However, there were no significant correlations between ROM and age. Thus, we speculate that the effect of predominantly osteoporosis to ROM in this study is less strong. A recent report indicated that serum oxidative stress markers were elevated in sarcopenia [23]. On the other hand, in this study, there were no cases that had pain recurrence (and required revision surgery) due to the recurrence of disc herniation or other segment disorders including adjacent segment degenerations within one-year follow-up. Generally, an adjacent segment degeneration after lumbar interbody fusion surgery mainly occurs at a long-term period of over 2 years after surgery [24]. Thus, the effect of other segment diseases to ROM levels is basically eliminated in this study. In addition, we excluded all the trauma cases including minor trauma or the cases complicated with rheumatoid arthritis or neurodegenerative diseases that may strongly affect the change of ROM levels. However, we could not completely exclude all the factors other than neurogenic oxidative stress. This is also a limitation of this study. Ultimately, further studies to investigate the correlation between ROM and bone mineral density or muscle volume using a body composition analyzer are indispensable. Fifth, the severity of neurological symptoms and selection of surgical procedure could be subject to bias. However, all surgeries in this study were performed using a posterior approach and a standardized surgical procedure to minimize damage to paraspinal muscles. Furthermore, it is impossible to standardize the duration of symptomatic period because this study uses human samples. To resolve this problem, further in vivo studies to standardize the duration of neuronal damage will be needed.

## 5. Conclusions

Moderate oxidative stress as measured by serum ROM levels was present in patients with lumbar degenerative disorders and levels of oxidative stress improved gradually rather than rapidly within 1 year of surgery. The clinical results suggested that neurogenic oxidative stress can be improved by surgery to treat lumbar degenerative disorders, and that levels of oxidative stress before surgery could reflect the severity of neurological damage. Moreover, residual oxidative stress can reflect poor surgical outcome. Further investigations including the parameters of antioxidant conditions and the relationship between oxidative stress markers and bone mineral density or muscle volume are indispensable to further clarify the relationship between levels of ROM and outcomes of surgery to treat lumbar degenerative disorders.

## Figures and Tables

**Table 1:** Relationship between reactive oxygen metabolite (ROM) levels and patient characteristics.

Number of cases	ROM before surgery (U.CARR)	Sex (male/female)	Age (years)	Cause of disorder (LSS/LDH)	Smoking (±)	Diabetes (±)	Surgical procedure (decompression/fusion)	Surgical time (min)	Bleeding during surgery (g)
80	388.5 ± 92.0 (193-755)	44/36	69.7 ± 13.1 (22-88)	64/16	10/70	19/61	44/36	196.6 ± 71.9 (86-398)	76.0 ± 77.6 (5-341)

ROM: reactive oxygen metabolites; LSS: lumbar spinal stenosis; LDH: lumbar disc herniation. Data are mean ± standard deviation (range).

**Table 2:** Levels of reactive oxygen metabolites (ROM) before and after surgery.

	Before surgery	3 months	6 months	1 year
ROM (U.CARR)	388.5 ± 92.0	380.3 ± 86.2	377.0 ± 77.2	367.6 ± 83.3^∗^
	(193-755)	(249-588)	(251-673)	(205-599)

ROM: reactive oxygen metabolites. Data are the mean ± standard deviation (range). ^∗^*p* < 0.05 for comparisons using a paired *t*-test (compared with ROM before surgery).

## Data Availability

Answer: no. Comment: the datasets used during the current study are available from the corresponding author on reasonable request.
